# Comparing efficacies of autologous platelet concentrate preparations as mono-therapeutic agents in intra-bony defects through systematic review and meta-analysis

**DOI:** 10.1016/j.jobcr.2023.08.007

**Published:** 2023-09-06

**Authors:** Shailesh Varshney, Anshuman Dwivedi, Vibha Dwivedi

**Affiliations:** a Department of Periodontology, School of Dental Sciences, Sharda University, Greater Noida, Uttar Pradesh, India; b Department of Stem Cells & Regenerative Medicine, Santosh University, Ghaziabad, Uttar Pradesh, India; c Department of Psychology, Himalayan Gharwal University, Uttarakhand, India

**Keywords:** Autologous platelet concentrates, Platelet rich fibrin, Platelet rich plasma, Plasma rich in growth factors, Intra-bony defects, Publication bias

## Abstract

**Aim:**

This systematic review and meta-analysis aimed to assess individually the regenerative potential of PRF (Platelet-rich Fibrin), PRP (Platelet-rich Plasma), and PRGF (Plasma Rich in Growth Factors) in comparison to OFD (Open Flap Debridement) alone for treating Intrabony defects, by calculating pooled effect sizes.

**Background:**

Relevant randomized controlled trials on humans were searched in PUBMED, COCHRANE CENTRAL, and GOOGLE SCHOLAR. Mean differences (MD) of Clinical Attachment level (CAL), Probing Pocket depth (PPD), and Defect Depth Reduction (DDR) between the Experimental and Control groups were used for calculating pooled effect sizes. Risk of bias was assessed using Cochrane's tool, and publication bias was evaluated through Funnel plots, Trim & Fill Method, and Rosenthal's Fail-Safe N Test.

**Review result:**

A total of 23 studies were identified for qualitative and quantitative analysis. These studies were categorized into PRF, PRP, and PRGF groups based on the type of APC used. PRF showed the highest CAL gain (1.60 mm, 95% CI = 0.963–2.232 mm, P < 0.001, I2 = 93.83%) and PPD reduction (1.76 mm, 95% CI = 1.056 to 2.446, P < 0.001, I2 = 96.05%). However, PRP exhibited the greatest DDR (3.42 mm, 95% CI = −13.67 to −20.50, P = 0.011, I2 = 87.27%). PRF and PRP demonstrated large effect sizes, while PRGF showed a small effect size.

**Conclusion:**

The use of PRF, PRP, and PRGF showed advantages in treating intrabony defects. However, caution is advised when interpreting the results due to heterogeneity and publication bias among the studies.

## Graphical abstract

**Figure:**
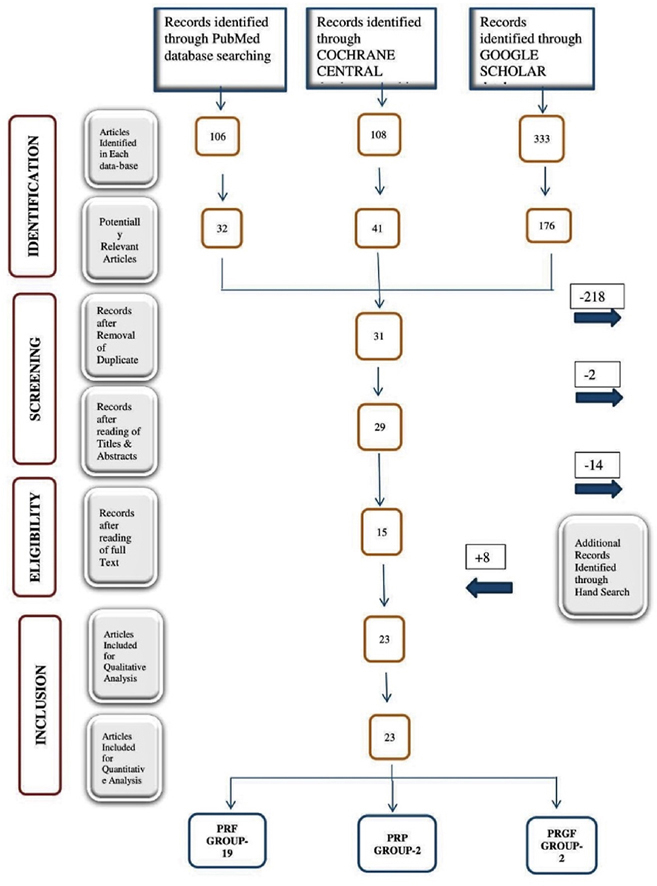

## 1 Introduction

Chronic periodontitis is an immuno-inflammatory condition that leads to progressive loss of attachment apparatus around affected teeth.^1^ Loss of attachment occurs as a result of destruction of periodontal structures namely cementum, alveolar bone and periodontal ligament. Though this condition is considered to be irreversible, regeneration of lost structures to restore the architecture and function of the periodontium has been the ultimate goal of many clinicians.^2^

Regeneration of lost periodontium is a complex process and clinicians need to aim at regenerating it as a single unit.^3^ Thus the lack of formation of any one of the three components of periodontium may lead to repair rather than regeneration.

Advances made in understanding the complex nature of periodontal wound healing have demonstrated the key role of growth factors (GFs) in periodontal regeneration.

During periodontal wound healing, the recruitment of locally derived progenitor cells occurs. These progenitor cells then differentiate into cementoblasts, periodontal ligament forming cells or bone-forming osteoblasts in the defect site. Polypeptide growth factors play a key role in migration, attachment, proliferation and differentiation of periodontal associated progenitor cells.^4^

Polypeptide Growth factors include an array of peptides such as insulin-like growth factor (IGF), vascular endothelial growth factor (VEGF), transforming growth factor (TGF)-, acidic and basic fibroblast growth factor (FGF), epidermal growth factor (EGF), platelet-derived growth factor (PDGF), and bone morphogenetic protein.

These peptides in the form of growth factors, perform various actions like stimulation, proliferation and differentiation of osteoblasts & gingival fibroblasts, and play a key role in the formation of extracellular matrix particularly Type 1 collagen, blood vessels, granulation tissues, during periodontal tissue healing.

Biologic agents like Autologous Platelet Concentrates (APCs) are potential regenerative agents containing polypeptide GFs. Various preparations of APCs are available for clinical use in periodontal regeneration.^5^ These include First Generation PCs like PRP (Platelet Rich Plasma) and PRGF (Plasma Rich in Growth Factors) whereas Second Generation PCs include PRF (Platelet Rich Fibrin). First Generation PCs use anticoagulants and activators for their preparations, are used in the form of liquid or Gels, take slightly longer time for their preparation, have a weaker fibrin network and release GFs up to 7 days. In Contrast, Second generation PCs do not use any anticoagulants, takes less time, has higher fibrin content in them and release GF's over a period of 7–14 days.^5^

To estimate the therapeutic benefits of the APCs in periodontal therapy, clinicians have compared them in various types of periodontal defects like intra-bony defects (IBDs), furcation defects, alveolar ridge preservation, during endosseous implant insertion, treatment of gingival recessions, increasing the width of attached gingiva in their Randomised Control Trials.

Among all, Intra-bony defects provide researchers with the best type of periodontal wounds to estimate the efficacy of above mentioned biologics (APCs) for the regeneration of both hard and soft tissues of the periodontium.

Many authors have conducted meta-analyses to estimate the efficacy of APCs in intra-bony defects. Notably among them include Castro et al.^6^ 2017, Del Fabbro et al.,^7^ 2011and Liang Chen et al.,^8^2021. But these authors in their meta-analysis have calculated the effect size based on combining either PRF^8^ or PRP^6^^,^^7^^,^^9^ with various types of bone grafts like bovine porous bone mineral (BPBM), demineralized freeze-dried bone allograft (DFDBA), nano-bone, autologous bone graft (ABG) or other biologics like Emdogain, Recombinant human platelet-derived growth factorBB (rhPDGF-BB), Bone Morphogenic Proteins (BMP) in intra-bony defects.

Thus none of the researchers has evaluated the effect size individually of each preparation of APCs i. e PRF, PRP and PRGF when used all alone i. e without combining them with bone grafts and/or biologics in Intra-bony defects.

Thus the aim of this evidence-based systematic review and meta-analysis (SRMA) is to estimate the regenerative potential of each APC preparations namely PRF, PRP and PRGF as monotherapeutic agents by comparing their effect size when used in OFD (Open Flap Debridement) versus OFD alone in the treatment of intra-bony defects.

## 2 Methodology

### 2.1 Search strategy

This Systematic review was conducted following the guidelines of PRISMA i.e Preferred Reporting Items for Systematic reviews^10^ and Meta-Analyses statement and the Cochrane Handbook for Systematic Reviews of Interventions.^11^ A comprehensive search was made in three databases namely Pubmed, Cochrane Central and Google Scholar using a combination of keywords and Controlled vocabulary. For searching these databases, terminologies were used combing Boolean Operators 'AND', 'OR' and 'NOT'. Following terms were used ("platelet concentrates" OR "platelets rich plasma" OR "PRP" OR "platelet rich fibrin" OR "PRF" OR "plasma rich in growth factors" OR "PRGF") AND ("intra-bony defects" OR "two-wall defects" OR "three wall defects") AND ("randomised control trials") NOT ("systematic reviews") NOT ("meta-analysis"). These terms were used in 'All Fields' during field search in databases. The search of articles was limited to the year 2021end, restricted to clinical trials involving human subjects and published in English language only. Cross-references of selected articles were also examined for the possibility of additional studies. Literature search was performed by two examiners. (SV, AD).

### 2.2 Research question

What is the adjunctive effect of APCs when compared to Open Flap Debridement (OFD) alone in the treatment of intra-bony defects in subjects with periodontitis?

### 2.3 PICO(ST) analysis

**Population (P)** - Periodontitis subjects having one or more Intra-bony defects diagnosed clinically as well as radiographically.

**Intervention (I)** - Treatment included the application of one of the three APCs i. e PRF or PRP or PRGF alone in intra-bony defects.

**Comparison (C)** - Treated Intra-bony Defects were compared with OFD alone.

**Outcome (O)** - Clinical Parameters that were assessed to evaluate the outcome were the difference in the Pocket Probing Depth (PPD) and Clinical or Relative Attachment Levels (CAL/RAL) whereas radiographic parameters included were changes in Defect Depth Reduction (DDR).

**Study (S)** – Clinical Trials conducted on Humans.

**Time (T) -** Studies included were from inception till December 2021.

### 2.4 Eligibility criteria

Study Groups from the selected studies were included if

1. They were part of Randomized Controlled Trials (RCTs)
2. APCs were used in the surgical treatment of Intra-bony defects.
3. Intervention group receiving APC was compared to an OFD Group.
4. They had systemically healthy subjects with no abnormal platelet counts.
5. Outcome (O) Parameters mentioned in the PICO(ST) format were analyzed.

Study Groups from the selected studies were excluded if

1. The Intervention Group receiving APC was compared to a Control Group receiving another APC preparation or APC mixed with any other regenerative material or biologic agent.
2. They were part of animal studies, case-control studies, case series or case reports.
3. Investigating any other oral surgical intervention like tooth extraction, implant therapy, treatment of jawbone defects, odontogenic cysts and periapical surgery.

### 2.5 Data extraction and study selection process

In the first Stage of the selection process, potentially relevant studies were chosen among the various articles identified from the aforementioned Data Bases, by the examiners. (AD, VD).

The next stage involved the screening of articles where duplicate records were removed after reading their Titles and Abstracts.

Studies following all the Inclusion and Exclusion criteria were found to be eligible after reading full texts of screened articles. From the eligible studies, study groups were identified for Qualitative and Quantitative analysis.

### 2.6 Data analysis

In this Meta-analysis, a comparison was done between (1) PRF + OFD versus OFD alone (2) PRP + OFD versus OFD alone and (3) PRGF + OFD versus OFD alone.

The effect size was calculated in each group using CAL and Pocket Probing Depth as Clinical Variables and Defect Depth Reduction as Radiological Variables.

For each variable, the Mean difference (MD) was estimated between the Experimental and the Control Group from the baseline to the final follow-up period.

Pooled weight Mean Difference for the outcome variables (parameters) was evaluated using Meta-essentials Software Version 1.5 (https://www.erim.eur.nl/research-support/meta-essentials/)

The difference in the effect size between the experimental and the control groups was represented graphically in the form of Forest Plots. The significance level for this meta-analysis model was set at 0.05. The chi-square (χ2) and *I*^*2*^ tests were used to calculate statistical heterogeneity among the included studies. When the heterogeneity among the studies was more (P ≥ 0.10 and I^2^ ≥ 50%), the random-effects model was used for data merging. On the contrary, when P < 0.10 and I^2^ ≤ 50%, the fixed effects models were used. Funnel plot, Trim & Fill Method and Rosenthal's Fail-Safe N test were used to evaluate publication bias among selected studies.

### 2.7 Risk of bias assessment

The risk of bias for the included studies was assessed by 2 reviewers (VD, SV) following the Criteria of Cochrane Handbook for Systematic Reviews of Interventions.^11^ Seven criteria were evaluated by the reviewers, which included (1) random sequence generation (selection bias), (2) allocation concealment (selection bias), (3) blinding of participants and personnel (performance bias), (4) blinding of outcome assessment (detection bias), (5) incomplete outcome data (attrition bias), (6) selective outcome reporting (reporting bias), and (7) other bias. Depending upon, the presence or absence of none, one or more, or all the criteria, a particular study was termed as having a 'Low', 'Unclear' or 'High' risk of bias.

## 3 Results

**Fig. 1:**
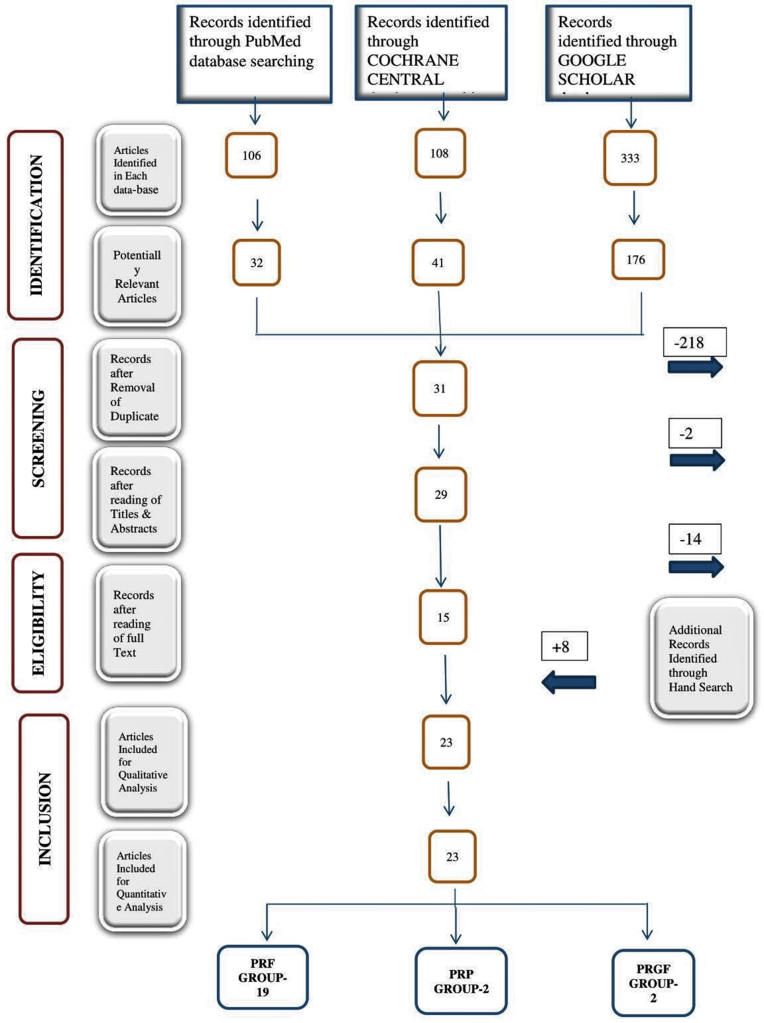
PRISMA flow chart.

A total of 547 studies were identified from the various electronic databases (Pubmed-106, Cochrane Central-108 and Google Scholar-333) using terminologies as described for the initial search strategies (Fig. 1). Among them, 249 potentially relevant studies were selected on the bases of their ‘Titles’. During the screening of articles, Titles and abstracts of studies were read and all duplicates were removed, leading to 29 eligible studies.

Full texts of 29 eligible studies were read thoroughly by the reviewers and thus 15 studies were found to be fulfilling all the inclusion and exclusion criteria. Additional 8 studies were also added by the reviewers, after cross-referencing the bibliographic records of 15 included studies.

Thus, finally, 23 studies were included for both qualitative and quantitative analysis.

### 3.1 Study characteristics

The general attributes of the 23 included studies have been summarized in Table 1. The included studies were divided into three groups i. e PRF, PRP and PRGF group depending on the type of APCs used in the Experimental groups. All the included studies were Randomised Control Trials conducted among humans diagnosed with either Chronic or Aggressive Periodontitis and having Intrabony defects. These Randomized Control Trials were having either Parallel arm study designs^12^, ^13^, ^14^^,^^19^, ^20^, ^21^^,^^23^^,^^24^^,^^26^^,^^28^^,^^32^ or split mouth.^15^^,^^16^^,^^22^^,^^25^^,^^27^^,^^30^^,^^31^^,^^33^ The pooled effect size was estimated between the Experimental Group being treated with OFD plus one of the three APCs and the Control Group which was treated with OFD alone. Primary outcome variables (Clinical parameters) included site specific measurement of CAL and PPD whereas Secondary Outcome variable (Radiographic parameter) included site specific measurement of Defect Depth Reduction (DDR). In the included studies, PPD was measured either using manual probes alone or customised acrylic stents for standardization of probing angulation and reproducibility of the placement of the manual probe. Attachment level was estimated as CAL, a direct measurement from CEJ to the base of the periodontal pocket or RAL (rCAL) as a distance between a fixed point on a customised acrylic stent to the base of the pocket. Radiographic parameters were measured using conventional IOPA through long cone paralleling technique. Also, in some studies, conventional radiographs were digitalised using software or RVG was used.

**Table 1:** General characterestics of the included studies.

PRF GROUP							
AUTHOR,YEAR	STUDY DESIGN	NUMBER AT PARTICIPANTS AT BASE LINE (END OF THE STUDY)	SUBJECTS	TOTAL STUDY GROUPS	DURATION OF THE STUDY	FOR QUANTATIVE ANALYSIS	
						CONTROL GROUP	EXPERIMENTAL GROUP
Sharma et al.^12^,2011	Randomized, controlled clinical trial, Parallel arm Study	42 patients, 69 sites (35 patients, 56 sites)	Chronic Periodontitis	1.Test Group:PRF + OFD. 2 Control Group: OFD alone	9 Months	Control Group: OFD alone	Test Group: PRF + OFD.
Thorat et al.^13^,2011	Controlled Clinical Trial, Parallel arm Study	40 patients, 40 sites (32 patients, 32 sites)	Chronic Periodontitis	1.Control Group: OFD alone 2. Test Group: OFD + PRF	9 Months	Control Group: OFD alone	Test Group: OFD + PRF
Pradeep et al.^14^,2012	Randomized, controlled clinical trial, Parallel Arm Study	54 patients, 98 sites (50 patients, 90 sites)	Chronic Periodontitis	1. Test Group 1: PRF + OFD 2. Test Group 2: PRP + OFD 3.Control Group: OFD alone	9 Months	.Control Group: OFD alone	Test Group 1: PRF + OFD
Rosamma et al.^15^,2012	Controlled Clinical Trial, Split Mouth Design	15 Patients, 30 sites (Same)	Chronic Periodontitis	1.Expermental Group: PRF + OFD 2.Control Group:OFD Alone	12 months	Control Group:OFD Alone	Experimental Group: PRF + OFD
Ajwani et al.,^16^2015	Randomized, longitudinal interventional study,Split-mouth Design	20 patients, 20 sites (Same)	Chronic Periodontitis	1.Test Side:OFD with PRF 2. Control Side: OFD alone	9 Months	Control Side: OFD alone	Test Side:OFD with PRF
Pradeep et al.,^17^2015	Randomized, controlled clinical trial, Parallel arm Study	136 patients, 136 sites (120 patients, 120 sites)	Chronic Periodontitis	1.OFD group 2.PRF Group 3.1% MF Group 4. OFD + PRF+ 1% MF Group	9 Months	OFD group	PRF Group
Chandradas et al.^18^,2016	Randomized, controlled clinical trial, Parallel arm Study	38 patients 38sites, (36 Patients, 36 sites)	Chronic Periodontitis	Group A:PRF + DBM Group B:PRF Group C: Control	9 Months	Group C: Control	Group **B**:PRF
Kanoriya et al.^19^,2016	Randomized, controlled clinical trial, Parallel arm Study	108 patients, 108 sites, (90 patients, 90 sites)	Chronic Periodontitis	1.Group 1: Access therapy alone 2.Group 2: Access therapy + PRF 3. Group 3:Access therapy + PRF +1% ALN	9 Months	Group 1:Access therapy alone	Group 2: Access therapy + PRF
Martande et al.^20^,2016	Randomized, controlled clinical trial, Parallel arm	110 patients, 110 sites (90 patients, 90 sites)	Chronic Periodontitis	1.Group 1: OFD + PRF 2.Group 2:OFD + PRF+1% ATV 3.Group 3: OFD alone	9 months	.Group 3: OFD alone	Group 1: OFD + PRF
Pradeep et al.^21^,2016	Randomized controlled trial, Parallel arm study	90 patients, 90 sites (Same)	Chronic Periodontitis	1.Group 1:OFD alone 2.Group 2:OFD + PRF 3. Group 3: OFD +1.2% RSV Gel	9 Months	.Group 1:OFD alone	Group 2:OFD + PRF
Arabacı et al.^22^,2017	A Randomized Split-Mouth Clinical Study	26 Patients, 52 sites (Same)	Chronic Periodontitis	1.OFD Group 2. OFD + PRF Group	9 Months	OFD Group	OFD + PRF Group
Bajaj et al.^23^,2017	Randomized, controlled clinical trial, Parallel Arm Study	19 patients, 59 sites (17 patients, 54 sites)	Aggressive Periodontitis	1.PRF Group:PRF + OFD 2. Control:OFD alone	9 Months	Control: OFD alone	PRF Group: PRF + OFD
Chatterjee et al.^24^,2017	Randomized, Comparative clinical study, Parallel Arm Study	38 patients, 90 sites (36 patients, 84 sites)	Chronic Periodontitis	1.Group 1:OFD alone 2. Group 2: PRF + OFD 3.Group 3: T-PRF + OFD alone	9 Months	Group 1:OFD alone	Group 2: PRF + OFD
Patel,^25^ et al., 2017	Randomized, controlled clinical trial, Split Mouth	20 Patients 40 sites (13 patients, 26 sites)	Chronic Periodontitis	1.Control Group: OFD alone 2. Test Group: OFD + PRF	12 Months	Control Group: OFD alone	Test Group: OFD + PRF
Pradeep et al.^26^,2017	Randomized controlled trial, Parallel arm study	62 patients, 104 sites (57 patients, 90 sites)	Chronic Periodontitis	1.Group 1:PRF group 2. Group 2: PRF + HA 3.Control Group: OFD alone	9 Months	Control Group: OFD alone	Group 1:PRF group
Thorat et al.^27^,2017	Randomized, controlled clinical trial, Split Mouth Design	18 patients, 36 sites (15 patients, 30 sites)	Aggressive Periodontitis	1.Test Side: OFD + PRF 2.Control Side:OFD alone	12 Months	Control Side:OFD alone	Test Side: OFD + PRF
Ustaoğlu, G et al.^28^,2020	Randomized, controlled clinical trial, Parallel Arm Study	45 Patients, 45 Sites (Same)	Chronic Periodontitis with Endo-Perio Lesions	1. Test Group 1: PRF + OFD 2. Test Group 2: PRP + OFD 3.Control Group: OFD alone	9 Months	Control Group: OFD alone	Test Group 1: PRF + OFD
Yajamanya et al.^29^,2020	Randomized controlled trial	Not Menrtioned, 60 sites (Same)	Chronic Periodontitis	1. Control Group:Access Flap Alone 2. Test Group 1: Access Flap + Perioglass 3. Test Group 2: Access flap + PRF	9 Months	Control Group:Acess Flap Alone	Test Group 2: Access flap + PRF
Pham TAV.^30^,2021	Randomized, controlled clinical trial, Split Mouth Design	32 patients, 96 sites (30 patients, 90 sites)	Chronic Periodontitis	1.Group 1:PRF + OFD 2. Group 2: GTR + OFD 3.Group 3: OFD alone	12 Months	Group 3: OFD alone	Group 1:PRF + OFD

PRP Group							
AUTHOR,YEAR	STUDY DESIGN	NUMBER AT PARTICIPANTS AT BASE LINE (END OF THE STUDY)	SUBJECTS	TOTAL STUDY GROUPS	DURATION OF THE STUDY	FOR QUANTATIVE ANALYSIS	
						CONTROL GROUP	EXPERIMENTAL GROUP
Agarwal et al.,.^31^2016	Comparative clinical trial, Split Mouth Design	10 patients, 30 sites (8 patients, 28 sites)	Chronic Periodontitis	1.Test Group A-PRP Alone. 2.Test Group B- PRP with DFDBA. 3. Control Group- OFD	12 Months	Control Group- OFD	Test Group A-PRP Alone.
Pradeep et al.^14^,2012	Randomized, controlled clinical trial, Parallel Arm Study	54 patients, 98 sites (50 patients, 90 sites)	Chronic Periodontitis	1. Test Group 1: PRF + OFD 2. Test Group 2: PRP + OFD 3.Control Group: OFD alone	9 Months	.Control Group: OFD alone	Test Group 2: PRP + OFD

PRGF Group							
AUTHOR,YEAR	STUDY DESIGN	NUMBER AT PARTICIPANTS AT BASE LINE (END OF THE STUDY)	SUBJECTS	TOTAL STUDY GROUPS	DURATION OF THE STUDY	FOR QUANTATIVE ANALYSIS	
						CONTROL GROUP	EXPERIMENTAL GROUP
Bojarpour et al.^32^,2018	Randomised Control Trial, Parallel arm study	5 patients, 20 sites (Same)	Chronic Periodontitis	1.Control Group:Group 1:OFD alone, 2 Treatment group 1 Group 2:OFD + Membranre 3. Treatment Group 2:Group 3:PRGF + Membrane	6 Months	Control Group:Group 1:OFD alone,	Treatment Group 2:Group 3:PRGF + Membrane
Sheethalan et al.^33^,2017	Randomized, controlled clinical trial, Split Mouth Design	14 Patients, 42 sites (12 Patients, 38 sites)	Chronic Periodontitis	1.Group 1:PRGF + GTR Membrane 2. Group 2: OFD + GTR Membrane	6 Months	Group 2: OFD + GTR Membrane	Group 1:PRGF + GTR Membrane

OFD- Open flap debridement, PRF-Platelet Rich Fibrin, PRP-Plasma Rich Proteins,HA-hydroxyapatite;, GTR-Guided Tissue Regeneration, ALN- Alendronate, ATV-Atorvastatin, RSV-Rosuvastatin**,** DBM-Demineralized Bone Matrix.

OFD- Open flap debridement, PRP-Plasma Rich Protein,DFDBA-Demineralized Freeze Dried Bone Allograft.

OFD- Open flap debridement, PRGF-Plasma Rich in Growth factors.

Out of the 23 included studies, 19 studies^12^, ^13^, ^14^, ^15^, ^16^, ^17^, ^18^, ^19^, ^20^, ^21^, ^22^, ^23^, ^24^, ^25^, ^26^, ^27^, ^28^, ^29^, ^30^ compared PRF with OFD alone whereas 2 studies each, compared PRP^14^^,^^31^ and PRGF^32^^,^^33^ with OFD alone.

Among all the selected studies for quantitative analysis, only 4 studies^13^^,^^16^^,^^18^^,^^33^ showed a statistically insignificant difference in PPD reduction between the Experimental and the Control group whereas for CAL measurement, 5 studies^12^^,^^14^^,^^16^^,^^17^^,^^33^ demonstrated statistically insignificant gain.

Among 23 selected studies, only 14 studies measured DD reduction in millimetres. Out of these, only 1 study^33^ showed statistically insignificant reduction in DD.

## 4 PRF studies (OFD + PRF V/S OFD alone)

### 4.1 CAL interpretation

**Fig. 2:**
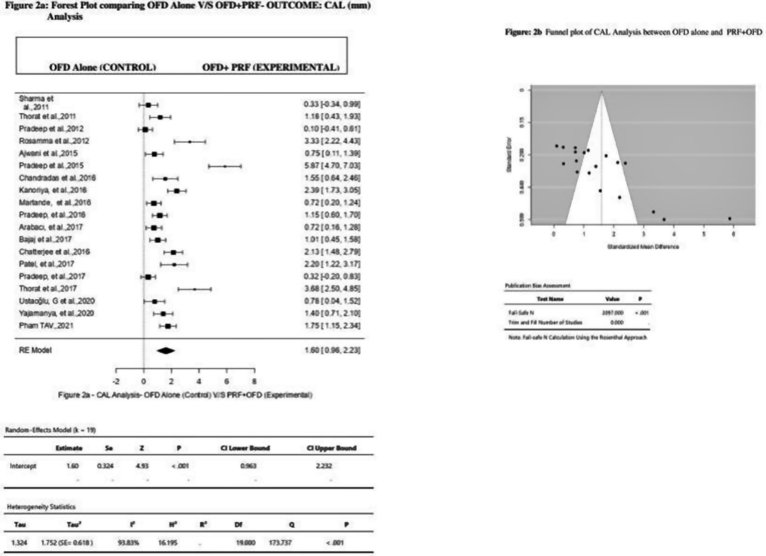
Forest plot and Funnel plot comparing OFD V/S OFD + PRF outcomes for CAL analysis.

Use of PRF was found to be advantageous, with gain in CAL more in the Experimental Group than the Control Group as assessed by the Random Effect Model. In this model, 19 studies were included for the evaluation. Overall, its effect on CAL was: MD 1.60 mm (Effect size), 95%CI = 0.963 to 2.232, P < 0.001, *I*^*2*^ = 93.83% (Fig. 2a) (Table 2). On visual examination, funnel plot appeared asymmetrical with uneven distribution of studies on both the sides of overall Effect Size (Fig. 2b). But Trim and fill method indicated the missing of 0 studies. Rosenthal Fail-Safe N test value of 2097 (Fig. 2b) (Table 3), was considered too high to give a false interpretation of the presence of Publication bias.

**Table 2:** Random – average effect size and heterogeneity statistics.

Parameters	Association	N	K	Hedges' g	95%CI	Q	*I**^2^*	p- Value
**CAL Gain**	**PRF + OFD v/s OFD alone**	*923*	*19*	1.60	0.963 to 2.232	173.73*	93.83*%*	<0.001
	**PRP + OFD v/s OFD alone**	*79*	*2*	*1.09*	*−12.21 to 14.38*	*11.43*	*91.25%*	*0.299*
	**PRGF + OFD v/s OFD alone**	*52*	*2*	*0.24*	*−0.08 to 1.30*	0.10*	*0.00%*	*0.004*
**PPD Reduction**	**PRF + OFD v/s OFD alone**	*923*	*19*	1.76	1.056 to 2.446	213.69*	96.05%	<0.001
	**PRP + OFD v/s OFD alone**	*80*	*2*	*0.88*	*−1.18 to 2.93*	0.49*	*0.00%*	*0.00*
	**PRGF + OFD v/s OFD alone**	*52*	*2*	*0.33*	*−2.35 to 3.01*	*0.61*	*0.00%*	*0.120*
**DDR**	**PRF + OFD v/s OFD alone**	*927*	*14*	2.36	0.88 to3.84	378.21*	98.43%	<0.001
	**PRP + OFD v/s OFD alone**	*79*	*2*	*3.42*	*−13.67–20.50*	7.85*	*87.27%*	*0.011*
	**PRGF + OFD v/s OFD alone**	*38*	*1*	*NA*	*NA*	*NA*	*NA*	*NA*

N = number of participants, k-no of effect sizes (studies) included for the analysis, CI = 95% Confidence interval, Q and *I*^*2*^ test of heterogeneity, Hedges'g = Random-average effect size * p < 0.05, NA- Not Applicable.

**Table 3:** Publication Bias statistics.

APC Groups	Parameters	K	Classic Fail-Safe N	Publication Bias
**PRF**	**CAL**	19	2097	False
	**PPD**	19	2745	False
	**DDR**	14	2709	False
**PRP**	**CAL**	2	5	True
	**PPD**	2	10	True
	**DDR**	2	53	False
**PRGF**	**CAL**	2	0	True
	**PPD**	2	0	True
	**DDR**	1	0	True

k- Number of studies, CAL-Clinical Attachment Level, PPD- Probing Pocket Depth, DDR- Defect, Depth Reduction.

### 4.2 PPD interpretation

**Fig. 3:**
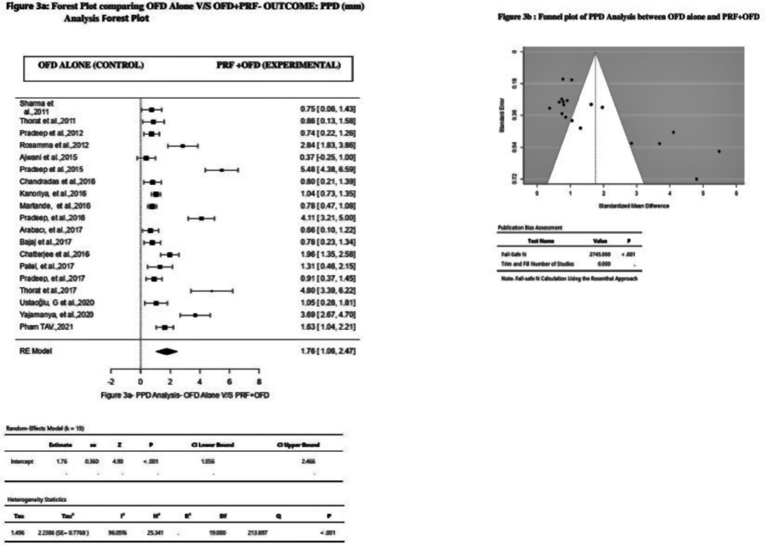
Forest plot and Funnel plot comparing OFD V/S OFD + PRF outcomes for PPD analysis.

The use of PRF was found to be advantageous, with more PPD reduction in the Experimental Group than in the Control Group as assessed by the Random Effect Model. In this model, 19 studies were included for the evaluation. Overall, its effect on PPD was: MD 1.76 mm (Effect size), 95%CI = 1.056 to 2.446, P < 0.001, *I*^*2*^ = 96.05% (Fig. 3a) (Table 2). On visual examination, the funnel plot appeared asymmetrical with uneven distribution of studies on both the sides of overall Effect Size, indicating the presence of definitive publication bias (Fig. 3b). But, the Trim and fill method indicated the missing of 0 studies. Rosenthal Fail-Safe N test value of 2745 (Fig. 3b) (Table 3), was considered too high to give a false interpretation of the presence of Publication bias. Thus this test indicated an absence of publication bias.

### 4.3 DDR interpretation

**Fig. 4:**
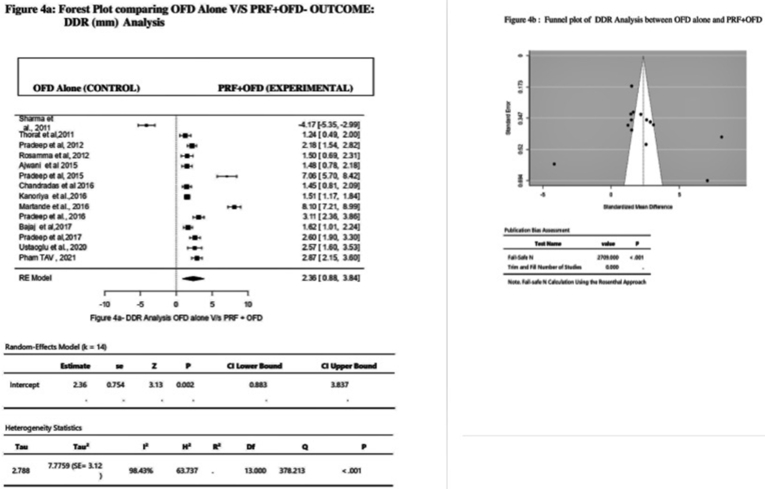
Forest plot and Funnel plot comparing OFD and OFD + PRF outcomes for DDR analysis.

DDR was found to be more in the Experimental Group when compared with the Control Group as assessed by the Random effect Model, indicating a positive effect of PRF on DDR. In this model, 14 studies were included for the evaluation. Overall, its effect on DDR was: MD 2.36 mm (Effect size), 95% CI = 0.88 to3.84, P < 0,001, *I*^*2*^ = 98.43% (Fig. 4a) (Table 2). On visual examination, Funnel plot appeared almost symmetrical with even distribution of studies on both sides of overall Effect Size (Fig. 4b). Also, the Trim and fill method indicated missing 0 studies with no adjustment in calculated Hedge's g and SE. Also, the Rosenthal Fail-Safe N test value of 2709 (Fig. 4b) (Table 2) was considered too high to give a false interpretation of the presence of Publication bias.

## 5 PRP studies (OFD + PRP V/S OFD alone)

### 5.1 CAL interpretation

Use of PRP was found to be advantageous, with gain in CAL more in the Experimental Group than the Control Group as assessed by the Random Effect Model. In this model, 2 studies were included for the evaluation. Overall, its effect on CAL was: MD 1.09 mm (Effect size), 95%CI = −12.21 to 14.38, P = 0.299, *I*^*2*^ = 91.25% (Table 2). Although the Funnel plot appeared symmetrical in distribution and the Trim and Fill test suggested no requirement for adding additional studies but Rosenthal Fail N Safe test value of 5 (Table 3) suggested the presence of publication bias.

### 5.2 PPD interpretation

The use of PRP was found to be advantageous, with more PPD reduction in the Experimental Group than in the Control Group as assessed by the Random Effect Model. In this model, 2 studies were included for the evaluation. Overall, its effect on PPD was: MD 0.88 mm (Effect size), 95%CI = −1.18 to 2.93, P = 0.00, *I*^*2*^ = 0.00 (Table 2)). Although the Funnel plot appeared symmetrical in distribution and the Trim and Fill test suggested no requirement for adding additional studies but Rosenthal Fail N Safe test value of 10 (Table 3) suggested the presence of publication bias.

### 5.3 DDR interpretation

DDR was found to be more in the Experimental Group when compared with the Control Group as assessed by the Random effect Model, indicating a positive effect of PRP on DDR. In this model, 2 studies were included for the evaluation. Overall, its effect on DDR was: MD 3.42 mm (Effect size), 95% CI = −13.67 to 20.50, P = 0.011, *I*^*2*^ = 87.27% (Table 2). Here the Funnel plot appeared symmetrical in distribution and the Trim and Fill test suggested no requirement for adding additional studies. Also, Rosenthal Fail N Safe test value of 58 (Table 3) suggested the presence of no publication bias.

## 6 PRGF studies (OFD + PRGF V/S OFD alone)

### 6.1 CAL interpretation

Use of PRGF was found to be advantageous, with gain in CAL more in the Experimental Group than the Control Group as assessed by the Random Effect Model. In this model, 2 studies were included for the evaluation. Overall, its effect on CAL was: MD 0.24 mm (Effect size), 95%CI = −3.81 to 3.67, P = 0.367, *I*^*2*^ = 0.00% (Table 2)). Although the Funnel plot appeared symmetrical in distribution and the Trim and Fill test suggested no requirement for adding additional studies but Rosenthal Fail N Safe test value of 0 (Table 3) suggested the presence of publication bias.

### 6.2 PPD interpretation

The use of PRGF was found to be advantageous, with more PPD reduction in the Experimental Group than in the Control Group as assessed by the Random Effect Model. In this model, 2 studies were included for the evaluation. Overall, its effect on PPD was: MD 0.33 mm (Effect size), 95%CI = −2.35 to 3.01, P = 0.12, *I*^*2*^ = 0.00% (Table 2)). Although the Funnel plot appeared symmetrical in distribution and the Trim and Fill test suggested no requirement for adding additional studies but Rosenthal Fail N Safe test value of 0 (Table 3) suggested the presence of publication bias.

### 6.3 Risk of bias assessment

Cochrane Collaboration tool was used to assess the risk of bias present within the included studies (Table 4). Authors of all the included studies have described the method of randomization used for the allocation of participants or the sites, into the various study groups. Whereas only three studies^18^^,^^22^^,^^33^ did describe the method of Allocation Concealment like the use of opaque envelopes. No other studies clearly mentioned the process of Allocation Concealment. All the studies were either single or double-blinded, Since all these RCTs were surgical interventions, the authors of 10 studies did not clearly describe whether the participants were blinded or not. Two studies^12^^,^^26^ did not mention the number of participants excluded during the follow-up period of the study whereas three studies^22^^,^^30^^,^^33^ did not mention this criterion clearly. No 'Reporting Bias' was seen in the included studies as the results and inferences made by the authors were as per the statistical valve observed in their calculations. Only two studies^15^^,^^28^ indicated the presence of 'Other Biases' where they failed to either describe or mention Inter or Intra examiner variability.

**Table 4:** Risk of Bias (ROB) of the included studies.

AUTHOR,YEAR	Random sequence generation (selection bias)	Allocation concealment (selection bias)	Blinding of participants and personnel (performance bias)	Blinding of outcome assessment (detection bias)	Incomplete outcome data (attrition bias)	Selective reporting (reporting bias)	Other bias
Sharma et al.^12^,2011	+	?	+	+	–	+	+
Thorat et al.^13^,2011	+	?	?	+	+	+	+
Pradeep et al.^14^,2012	+	?	+	+	+	+	+
Rosamma et al.^15^,2012	+	?	?	+	+	+	_
Ajwani et al.^16^,2015	+	?	?	+	+	+	+
Pradeep et al.^17^,2015	+	?	+	+	+	+	+
Chandradas et al.^18^,2016	+	+	?	+	+	+	+
Kanoriya et al.^19^,2016	+	?	+	+	+	+	+
Martande et al.^20^,2016	+	?	+	+	+	+	+
Pradeep et al.^21^,2016	+	?	+	+	+	+	+
Arabacı et al. 22.,2017	+	+	+	+	?	?	+
Bajaj et al.^23^,2017	+	?	+	+	+	+	+
Chatterjee et al.^24^,2017	+	?	?	?	+	+	+
Patel et al.^25^,2017	+	?	+	+	+	+	+
Pradeep et al.^26^,2017	+	?	+	+	_	+	+
Thorat et al.^27^,2017	+	?	?	+	+	+	+
Ustaoğlu, G et al.^28^,2020	+	?	?	+	+	+	_
Yajamanya et al.^29^,2020	+	?	?	?	?	+	+
Pham TAV.^30^,2021	+	?	+	+	+	+	+
Agarwal et al.,.^31^2016	+	?	+	+	+	+	+
Bojarpour et al.^32^,2018	+	?	?	?	?	+	+
Sheethalan et al.^33^,2017	+	+	+	+	+	+	+

+ (NO RISK) ? (RISK) − (HIGH RISK).

Overall, after assessing the seven criteria of the Cochrane Collaboration Tool, 4 studies^12^^,^^15^^,^^26^^,^^28^ were graded as having a High Risk of Bias, 3 studies^24^^,^^29^^,^^32^ as having Moderate Risk of Bias and the rest of the studies were a Low risk of Bias.

## 7 Discussion

The present Systematic Review and Meta-analysis was conducted to evaluate the efficacy of three different biologic preparations namely PRF, PRP and PRGF as a mono-therapeutic agents. In the treatment of Intra-bony periodontal defects. Their effect was evaluated by measuring observed changes in both Clinical (CAL & PPD) and Radiographic (DDR) parameters.

Looking into the results, most CAL gain was observed when intra-bony defects were treated with OFD combined with PRF with overall effect size of 1.60 mm, 95%CI = 0.963–2.232 mm, P < 0.001, *I*^*2*^ = 93.83% (Fig. 2a) (Table 2) rather than PRP (Effect size = 1.09 mm, 95%CI = −12.21 to 14.38 mm, P = 0.299, *I*^*2*^ = 91.25%) (Table 2) and PRGF (Effect size = 0.24 mm, 95%CI = −0.08 to 1.30, P = 0.004, *I*^*2*^ = 0.00%). (Table 2).

Similarly, the most PPD reduction was observed when intra-bony defects were treated with OFD combined with PRF with overall effect size of 1.76 mm, 95%CI = 1.056 to 2.446, P < 0.001, *I*^*2*^ = 96.05% (Fig. 3a) (Table 2) rather than PRP (Effect size = 0.88 mm, 95%CI -1.18 to 2.93, P = 0.00, *I*^*2*^ = 0.00) (Table 2) and PRGF. (Effect size = 0.33 mm, 95%CI -2.35 to 3.01, P = 0.120, *I*^*2*^ = 0.00) (Table 2).

But with respect to DDR, highest reduction was seen with PRP with overall effect size of 3.42 mm, 95% CI = −13.67 to −20.50, P = 0.011, *I*^*2*^ = 87.27% (Table 2) rather than PRF (Effect size = 2.36 mm, 95%CI 0.88 to3.84, P < 0.001, *I*^*2*^ = 98.43%) (Fig. 4a) (Table 2). Effect size of PRGF for DDR parameter could not be calculated due to the lack of minimum number of required studies.

In our Meta-analysis, the overall effect size was calculated in terms of Hedges' g which uses the Mean differences between the Experimental and the Control Groups of the included Randomized Control Trials. According to Cohen^34^ any value of Hedges' g above 0.8 is considered to be a large effect size suggesting intervention done has favourable results in the experimental group compared to the control group.

Thus the average weighted effect size obtained from the secondary data of included studies indicated that the use of PRF and PRP are significantly effective in gain in CAL (1.60 mm, 1.09 mm respectively) and reduction in PPD (1.76 mm, 0.88 mm respectively) and DD (2.36 mm, 3.42 mm respectively.) in intra-bony defects. PRGF has also been found to be effective but to a smaller extent.

By comparing the three biologic agents, authors have tried to establish superiority of one agent upon another which have gained increased popularity in recent times, in the field of periodontal regeneration. Among them, PRF which is a second generation APC, have shown to have superior outcomes when compared to PRP and PRGF. Studies have shown that PRF forms a dense fibrin network and has an extended period of growth factors release.^35^ Also with the use of anticoagulants in preparation of PRP and PRGF may affect the release of GFs from platelets.^36^^,^^37^

Moreover, further analysis of the obtained data suggests the existence of high-level heterogeneity (*I*^*2*^) among studies, ranging from 87.27% to 98.43%% (Table 2). Thus the results of this analysis have to be considered cautiously. Heterogeneity among studies could be due to variability in the method of preparation of APCs, measurement of parameters like PPD, CAL or DDR, the concentration of platelets cells among participants, type of intra-bony defects, type of periodontitis, study designs of RCTs etc. Another causal factor for heterogeneity could be the g-forces value of the centrifuge. During centrifugation, value of g-forces vary with tube angulation, rotor size and/or bucket size/type. In order to standardize this force, one need to use a centrifugation machine designed with same centrifugation radius, with the same tube angulation, fabricated with same composition, filled with blood to the same level and using the same centrifugation tubes.^38^

Many of these factors could be standardized easily in near future whereas few other factors would require extensive research. But the fact is that, the included studies in this analysis have been conducted over past 2 decades and variability factors related to g forces and others still remain in the included studies. Since many factors are involved in causing heterogeneity during the conduction of RCTs and it is not possible to negate them through subgroup or moderator analysis. As a result, outcome data in this analysis shows high level of heterogeneity. So in this meta-analysis authors have chosen the values obtained in Random Model rather than Fixed Model.

Publication bias assesses whether the systematically or comprehensively searched literature or published studies, represent the completed research in the field or not. It represents the number of unpublished studies with insignificant results which are to be included in the quantitative analysis to obtain a true effect size. Thus it is also an indirect method to check the reliability of the obtained data.

Funnel plot is a subjective method to assess the existence of publication bias by observing the symmetry of the distribution of studies around the pooled effect size. Whereas Rosenthal's Classic Fail-Safe N and Trim & Fill Method are objective means for locating publication bias.

Though the Funnel plots appeared asymmetrical for all but one parameter assessed i. e DD reduction (Fig. 4b) during PRF analysis, whereas Rosenthal Fail-Safe N test suggested the presence of no publication bias in terms of CAL, PPD and DDR (Table 3) in those group of studies. On the other hand, the aforementioned tests showed the presence of Publication bias in studies included under PRP and PRGF categories (Table 3) where Funnel plots appeared symmetrical.

Recent systematic reviews and meta-analyses have also reviewed the beneficial effects of APCs in intra-bony defects comparing OFD + APCs and OFD alone. APCs that evaluated were PRF and PRP.^6^^,^^8^^,^^9^ PRGF was never evaluated in these types of interventions. More ever, one of the author^39^ did try to evaluate the beneficial effect of PRGF in intra-bony defects but could not do so, due to a lack of studies related to that category.

Thus in this meta-analysis, we have tried to compare APCs of all the three types with an increased number of studies compared to the previous meta-analysis following a high level of evidence and limited risk of bias.

S Panda et al.^39^ in 2014, evaluated the effect of PRF in intra-bony defects among 4 studies and reported significant improvement in terms of CAL gain, PD reduction and radiographic bone fill %.In the same meta-analysis, they reported significant improvement in all the aforementioned parameters with the use of PRP too in intra-bony defects.

Lang Chen et al.^8^ in 2020, reported pooled effect size of 1.24mm of CAL and 1.18mm of PD reduction among 14 studies evaluating the effect of PRF in intra-bony defects. Similarly, they also evaluated IBD reduction among 11 studies with a pooled effect size of 1.81 mm.

Also, Del Flabrro et al.,^7^ 2018 in their SRMA of Cochrane database, reported pooled size effect of 1.29mm of PD reduction and 1.47mm of CAL gain among 12 studies with the use of PRF in intra-bony defects with a moderate level of heterogeneity and a significant p-value.

Comparing our results with some of the similarly conducted meta-analyses shows almost the same results but with a slightly larger pooled effect size along with a high level of heterogeneity. A larger pooled effect size gained could be due to the inclusion of more studies compared to previously conducted meta-analyses.

It is also observed that in our meta-analysis, there were more studies included in the PRF group compared to PRP and PRGF groups. Thus it appears PRF to be the most preferred biologic agent for researchers in the management of Intra-bony defects. This could be due to its low cost, ease and less time of preparation.

A few of the limitations of our meta-analysis have been a failure to include unpublished data or grey literature. Our literature search included electronic databases and a few hand search studies. This limitation could have led to publication bias. Also, our included studies demonstrated a high level of heterogeneity (almost 90%) which could question the reliability of the results obtained. Reasons for heterogeneity could be analyzed through subgroup or moderator analysis and could be reduced by increasing the sample size. Most of the studies included in our Quantitative analysis had small to moderate sample sizes.

## 8 Conclusion

As emphasized by a few authors^7^^,^^39^ there was a need to evaluate the effect of PRF and PRGF in intrabony defects. We provided our readers with clinical evidence of the true effects of APCs over OFD alone without any confounding factors like regenerative techniques. This would certainly help in guiding clinicians in deciding to choose an appropriate biologic agent in the field of periodontal regeneration. Though the use of PRF has shown beneficial effects conclusively not only in the current but also in the previously concluded meta-analyses, but PRP and PRGF have also been found to be beneficial. Thus one could also expect to have synergetic effect with significant improvement in both clinical and radiographic parameters when these biologic agents are used in combination therapy with various types of bone grafts.

Certainly, there is a lack of literature with similar methodologies with regards to PRP and PRGF application in intra-bony defects. Thus there is a need to conduct more standardized clinical trials in this regard, to overcome the issues of heterogeneity and publication bias, and so as to compare these APCs on a level playing field.

## 9 Formatting of funding sources

This research did not receive any specific grant from funding agencies in the public, commercial, or not-for-profit sectors.

## Declaration of competing interest

None
